# Prognosis of lymphotropic invasive micropapillary breast carcinoma analyzed by using data from the National Cancer Database

**DOI:** 10.1186/s40880-019-0406-4

**Published:** 2019-10-21

**Authors:** Gary D. Lewis, Yan Xing, Waqar Haque, Tejal Patel, Mary Schwartz, Albert Chen, Andrew Farach, Sandra S. Hatch, E. Brian Butler, Jenny Chang, Bin S. Teh

**Affiliations:** 1 Department of Radiation Oncology, University of Arkansas for Medical Sciences, Little Rock, AR 72205 USA; 2 Department of Medicine, Houston Methodist Hospital, Houston, TX 77030 USA; 3 Department of Radiation Oncology, Houston Methodist Hospital, Houston, TX 77030 USA; 4 Department of Pathology and Genomic Medicine, Houston Methodist Hospital, Houston, TX 77030 USA; 5 Department of Radiation Oncology, Baylor College of Medicine, Houston, TX 77030 USA; 6 Department of Radiation Oncology, The University of Texas Medical Branch, Galveston, TX 77555 USA

**Keywords:** Breast cancer, Invasive micropapillary carcinoma, Radiotherapy, Surgery, Survival, Hormone receptor

## Abstract

**Background:**

Invasive micropapillary carcinoma (IMPC) is an uncommon subtype of breast cancer. Previous studies of this subtype demonstrated a higher propensity for lymph node metastases as compared with invasive ductal carcinoma (IDC). The purpose of the present study was to determine the clinical characteristics, outcomes, and propensity for lymph node metastasis of patients with IMPC of the breast recorded in the National Cancer Database (NCDB).

**Methods:**

Records of patients with IMPC diagnosed between 2004 and 2014 were retrieved from the NCDB. Log-rank test was performed to evaluate associations of clinical characteristics with overall survival (OS). Cox proportional hazards model was used to determine variables associated with OS.

**Results:**

Overall, 2660 patients with IMPC met the selection criteria; the 5-year OS rate was 87.5% and 24.9% of patients had nodal involvement at presentation. Patients with ≥ 4 positive lymph nodes had shorter OS than node-negative patients, whereas patients with 1–3 positive nodes had similar OS to node-negative patients. Age < 65 years, receipt of radiotherapy, and estrogen receptor positivity were also associated with prolonged OS. The benefit of radiotherapy was limited to IMPC patients undergoing lumpectomy; there was no benefit for the patients undergoing mastectomy (regardless of nodal positivity/negativity).

**Conclusions:**

Favorable prognostic factors of IMPC patients included age < 65 years, < 4 positive lymph nodes, receipt of radiotherapy, and estrogen receptor positivity. The results presented herein suggest a survival benefit associated with radiotherapy in IMPC treatment, though this may be limited to the patients treated with lumpectomy.

## Background

Invasive micropapillary carcinoma (IMPC) of the breast is an uncommon variant of breast cancer that was first described in 1980 [1]. Histologically, this subtype appears as tumor cells arranged in small solid fragments or tubules with small or obliterated lumina, which appear as micropapillae without central fibrovascular cores [2]. These micropapillae are surrounded by clear stromal spaces not lined by endothelial cells, giving it an appearance similar to retraction artifact [3]. IMPC constitutes less than 2% of all invasive breast cancers, although 3%–6% of invasive breast cancers were reported to have a focal micropapillary growth pattern [4].

Previous studies demonstrated that IMPC was associated with lymphovascular invasion and a higher propensity for lymph node metastases than invasive ductal carcinoma (IDC) and other invasive subtypes of breast cancer [5–8]. It has been thought that, due to the lymphotropic nature of IMPC, these patients experience worse overall outcomes than those with IDC. The National Cancer Database (NCDB) is a national hospital-based cancer registry that is co-sponsored by the American College of Surgeons (ACoS) and the American Cancer Society. It houses data from more than 1500 hospitals with ACoS-accredited cancer treatment programs, accounting for almost 70% of all newly diagnosed cancer cases in the United States [9–14]. In this study, we aimed to analyze the survival outcomes of IMPC patients recorded in the NCDB.

## Patients and methods

### Patient selection

Records of patients with biopsy-proven IMPC diagnosed between January 2004 and December 2014 were retrieved from the NCDB. Diagnosis was made according to the International Classification of Disease for Oncology, third edition (ICD-O-3), code 8507. This study only included patients with American Joint Committee on Cancer (AJCC, 7th edition) stage cT1-4N0-3M0 pure IMPC and complete records regarding surgical therapy and radiotherapy.

### Prognosis analysis

Data of patient’s age, race, sex, Charlson–Deyo comorbidity score, histologic grade, estrogen receptor (ER) status, progesterone receptor (PR) status, human epidermal growth factor receptor 2 (HER2) status (only available for patients diagnosed between 2010 and 2014), TNM stage, number of positive lymph nodes, type of surgical resection, and the receipt of external beam radiotherapy (EBRT), chemotherapy, and hormonal therapy were collected.

Univariate analysis evaluated factors associated with overall survival (OS); subsequently, Cox multivariate analysis included variables that were statistically significant with a *P* value of < 0.05. OS was defined as the duration from the date of diagnosis to the date of last follow-up and was assessed using the Kaplan–Meier method. Patients were censored at the data of either death or the last follow-up. Only patients with complete data for the parameters of interest were included in the final analysis. Statistical analyses were performed using Stata/SE version 10 for Windows (StataCorp, College Station, TX, USA). A *P* value < 0.05 was considered significant.

## Results

### Patient characteristics

**Fig. 1:**
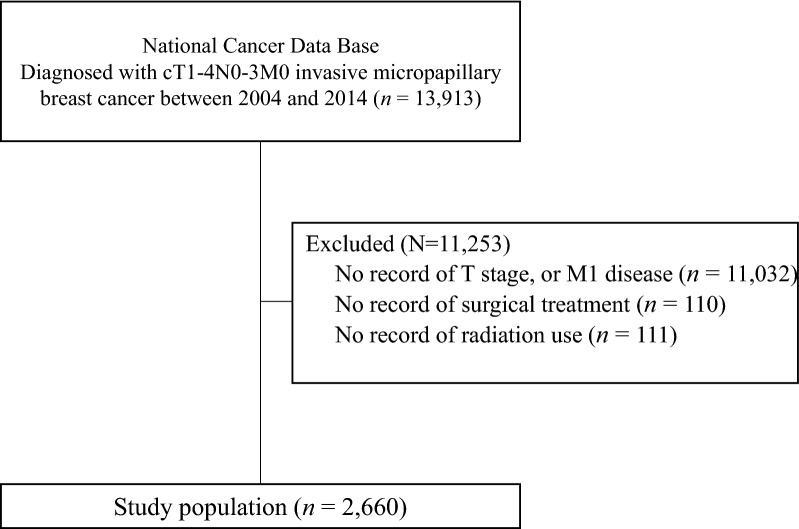
Diagram of selecting records of patients with invasive micropapillary breast cancer from the National Cancer Database

Overall, as shown in Fig. 1, a total of 2660 patients met the selection criteria. Median follow-up was 40 months (range 0.5–137 months). The median age of diagnosis was 60 years (range 19–90 years). Complete patient characteristics are summarized in Table 1.

**Table 1:** Characteristics of the 2660 patients with invasive micropapillary breast cancer

Characteristic	All patients [cases (%)]
Total	2660
Age	
≤ 50 years	564 (21.2)
51–64 years	1031 (38.8)
≥ 65 years	1065 (40.0)
Race	
White	2169 (81.5)
African American	351 (13.2)
Others	140 (5.3)
Sex	
Female	2607 (98.0)
Male	53 (2.0)
Charlson–Deyo comorbidity score	
0	2210 (83.1)
1	362 (13.6)
≥ 2	88 (3.3)
Clinical T stage	
T1	1655 (62.2)
T2	738 (27.7)
T3	184 (6.9)
T4	83 (3.1)
Clinical N stage	
N0	1998 (75.1)
N1	532 (20.0)
N2	81 (3.0)
N3	49 (1.8)
Surgery	
Lumpectomy	1281 (48.2)
Mastectomy	1379 (51.8)
Lymph nodes involved	
0	1243 (46.7)
1–3	689 (25.9)
≥ 4	501 (18.8)
Unknown	227 (8.5)
Histologic grade^a^	
1	196 (7.4)
2	1333 (50.1)
3	971 (36.5)
Not reported	160 (6.0)
ER status	
Positive	2327 (87.5)
Negative	291 (10.9)
Unknown	42 (1.6)
PR status	
Positive	2112 (79.4)
Negative	498 (18.7)
Unknown	50 (1.9)
HER2 status	
Positive	397 (14.9)
Negative	1498 (56.3)
Unknown	765 (28.8)
Tumor markers	
ER+ HER2−	1402 (52.7)
ER+ HER2+	297 (11.2)
ER− HER2+	99 (3.7)
ER− HER2−	95 (3.6)
Not reported	767 (28.8)
Radiotherapy	
Yes	1592 (59.8)
No	1068 (40.2)
Chemotherapy	
Yes	1273 (47.9)
No	1024 (38.5)
Not reported	363 (13.6)
Hormonal therapy	
Yes	1979 (74.4)
No	501 (18.8)
Not reported	180 (6.8)

*ER* estrogen receptor, *PR* progesterone receptor, *HER2* human epidermal growth factor receptor 2

^a^Well, moderately, and poorly differentiated/undifferentiated tumors were classified into histologic grades 1, 2, and 3, respectively

At presentation, 662 (24.9%) patients had nodal involvement. In terms of histologic grade, 2304 (86.6%) patients had grade 2 or 3 disease, only 196 (7.4%) patients had grade 1 disease. In terms of biomarker status, 2327 (87.5%) had ER-positive disease, 2112 (79.4%) had PR-positive disease, and 397 (14.9%) had HER2-positive disease. Unfortunately, 765 (28.8%) patients had unknown HER2 status. Of the patients with complete biomarker status, a majority had hormone receptor-positive, HER2-negative disease.

In terms of surgery, 1281 (48.2%) patients underwent lumpectomy, and 1379 (51.8%) underwent mastectomy. Overall, 1592 (59.9%) patients received EBRT, 1979 (74.4%) received hormonal therapy, and 1273 (47.9%) received chemotherapy.

### Outcomes and prognostic factors

**Fig. 2:**
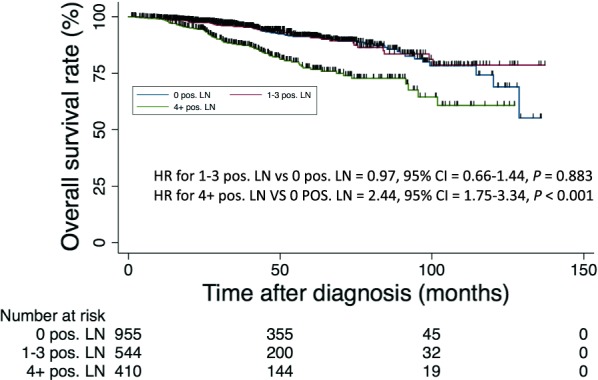
Kaplan–Meier overall survival curves of patients with invasive micropapillary breast cancer stratified by number of positive lymph nodes

At a median follow-up of 4 years (interquartile range 3.2–7.4 years), the 5-year OS rate was 87.5% (95% confidence interval [CI] 85.6%–89.4%). Univariate analysis showed that patients with ≥ 4 positive lymph nodes had shorter OS than patients with node-negative disease (hazard ratio [HR], 2.44; 95% CI 1.75–3.40; *P* < 0.001). However, those with 1–3 positive nodes had an OS similar to patients with node-negative disease (Fig. 2). As presented in Table 2, other indicators of poor prognosis on univariate analysis included age ≥ 65 years (HR, 1.93; 95% CI 1.33–2.81; *P* = 0.001), Charlson–Deyo comorbidity score = 1 (HR, 1.87; 95% CI 1.31–2.67; *P* = 0.001), Charlson–Deyo comorbidity score ≥ 2 (HR, 3.35; 95% CI 1.90–5.91; *P* < 0.001), omission of radiotherapy (HR, 1.76; *P* < 0.001), mastectomy (HR, 1.37; 95% CI 1.04–1.81; *P* = 0.025), ER-negative disease (HR, 2.24; 95% CI 1.63–3.11; *P* < 0.001), and lack of hormonal therapy use (HR, 1.53; 95% CI 1.13–2.05; *P* = 0.005). On multivariate analysis, factors associated with short OS included age > 65 years, Charlson–Deyo comorbidity score of 1 or ≥ 2, stage T2–4, stage N2, omission of radiation therapy, ER-negative disease, PR-negative disease, or ≥ 4 metastatic lymph nodes (*P* < 0.05 for all).

**Table 2:** Prognostic factors for overall survival of patients with invasive micropapillary breast cancer

Characteristic	Univariate analysis			Multivariate analysis		
	HR	95% CI	*P* value	HR	95% CI	*P* value
Age						
≤ 50	1.000			1.000		
51–64	0.783	0.515–1.193	0.255	0.899	0.585–1.382	0.628
≥ 65	1.929	1.327–2.805	0.001	2.494	1.657–3.752	< 0.001
Race						
White	1.000			–	–	–
African American	1.001	0.667–1.503	0.995	–	–	–
Other	0.591	0.277–1.257	0.172	–	–	–
Sex						
Female	1.000			–	–	–
Male	1.130	0.465–2.740	0.787	–	–	–
Charlson–Deyo comorbidity score						
0	1.000			1.000		
1	1.871	1.311–2.671	0.001	1.513	1.049–2.183	0.027
≥ 2	3.349	1.898–5.910	< 0.001	2.200	1.217–3.978	0.009
Clinical T stage						
1	1.000			1.000		
2	2.052	1.507–2.794	< 0.001	1.888	1.340–2.660	< 0.001
3	2.345	1.504–3.656	< 0.001	2.230	1.314–3.784	0.003
4	3.649	2.222–5.993	< 0.001	2.851	1.602–5.073	< 0.001
Clinical N stage						
0	1.000			1.000		
1	1.546	1.122–2.130	0.008	1.194	0.771–1.850	0.426
2	3.832	2.390–6.147	< 0.001	2.009	1.099–3.676	0.024
3	1.825	0.852–3.908	0.122	0.925	0.292–2.184	0.859
Surgery						
Lumpectomy	1.000			1.000		
Mastectomy	1.373	1.040–1.812	0.025	1.027	0.751–1.404	0.869
Radiotherapy						
Yes	1.000			1.000		
No	1.755	1.336–2.307	< 0.001	2.344	1.711–3.210	< 0.001
Histologic grade						
Well differentiated	1.000			–	–	–
Moderately differentiated	0.981	0.558–1.723	0.946	–	–	–
Poorly differentiated/undifferentiated	1.457	0.831–2.552	0.189	–	–	–
Not reported	0.721	0.291–1.788	0.481	–	–	–
ER status						
Positive	1.000			1.000		
Negative	2.245	1.629–3.113	< 0.001	2.804	1.827–4.305	< 0.001
Unknown	0.704	0.260–1.906	0.490	0.585	0.211–1.626	0.304
PR status						
Positive	1.000			1.000		
Negative	1.6501	1.223–2.227	0.001	1.586	1.166–2.156	0.003
Unknown	0.731	0.298–1.788	0.492	0.825	0.335–2.045	0.677
HER2 status						
Positive	1.000			–	–	–
Negative	1.021	0.604–1.728	0.937	–	–	–
Unknown	0.857	0.503–1.460	0.570	–	–	–
Chemotherapy						
Yes	1.000			–	–	–
No	0.914	0.679–1.230	0.552	–	–	–
Not reported	1.314	0.874–1.977	0.189	–	–	–
Hormonal therapy						
Yes	1.000			1.000		
No	1.526	1.134–2.054	0.005	0.789	0.538–1.158	0.227
Not reported	1.296	0.745–0.254	0.358	1.013	0.567–1.810	0.965
Lymph nodes involved						
0	1.000			1.000		
1–3	0.971	0.656–1.438	0.883	1.087	0.700–1.687	0.711
4 or more	2.438	1.750–3.398	< 0.001	2.292	1.413–3.719	0.001
Unknown	1.823	1.174–0.828	0.007	1.507	0.962–2.361	0.073

*OS* overall survival, *HR* hazard ratio, *CI* confidence interval, *ER* estrogen receptor, *PR* progesterone receptor, *HER2* human epidermal growth factor receptor 2

**Fig. 3:**
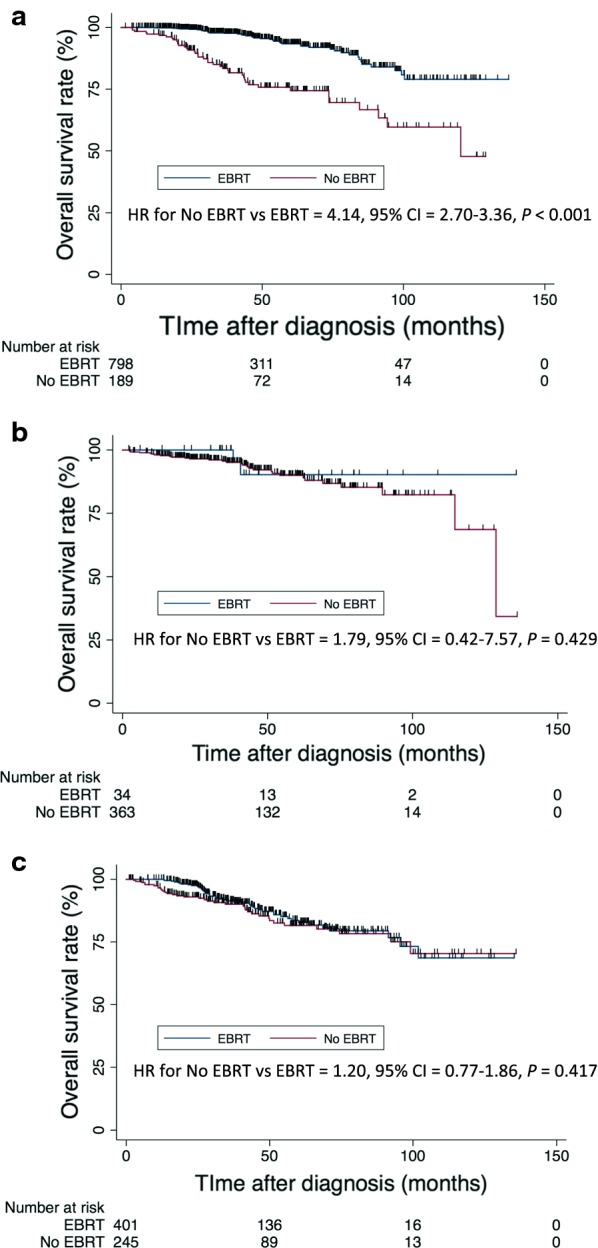
Kaplan–Meier overall survival curves of patients who underwent surgery with or without external beam radiotherapy. **a** Patients receiving lumpectomy; **b** patients with negative lymph nodes receiving mastectomy; and **c** patients with positive lymph nodes receiving mastectomy

Figure 3 represents Kaplan–Meier curves comparing overall survival of patients who underwent surgery either with or without radiotherapy. A longer OS was associated with radiotherapy among patients receiving lumpectomy, but such association was not observed among patients with either positive or negative nodal disease receiving mastectomy.

## Discussion

IMPC is a rare variant of breast cancer, making it difficult to study. As a result, using a large national database such as the NCDB allows for analysis using a large number of patients to help inform treatment management decisions. Our findings indicated that EBRT was associated with prolonged OS in IMPC patients undergoing lumpectomy but not for patients undergoing mastectomy. Additional poor prognostic factors for OS included older age, extensive lymph node involvement, and ER-positive disease.

The findings of this study are in accordance with those in the literature (Table 3) in that there is a higher rate of lymph node involvement seen in IMPC compared to the rate seen in IDC in previous studies. Because a higher rate of lymph node involvement and/or higher number of metastatic lymph nodes confers a higher N stage, it has been presumed that IMPC patients have worse survival outcomes than IDC patients. However, despite this higher propensity for lymph node involvement with IMPC than with IDC, we found that the 5-year OS rate of IMPC patients in our analysis was similar to the historical 5-year OS rate of patients with IDC reported in previous literature, which is in accordance with an analysis of a large group of IMPC patients using the Surveillance, Epidemiology, and End Results (SEER) database [3, 15].

**Table 3:** Literature review on invasive micropapillary carcinoma of the breast

Study	Cases	Age (years)^a^	ER+ (%)	PR+ (%)	HER2+ (%)	RLN+ (%)	DSS (%)	OS (%)	Follow-up duration^a^
NCDB (current study)	1818	60	84.3	73.9	19.4	55.2	–	87.5 at 5 years	4 years
Kim et al. [2]	38	47.3	19.4	19.4	–	78.9	–	–	–
Chen et al. [3]	624	61.7	84.8	69.9	–	52.9	92 at 5 years	84 at 5 years	33.0 months
Yu et al. [5]	267	47	66.3	66.3	28.8	62.9	–	97.7 at 5 years	59 months
Chen et al. [6]	95	58.9	83.2	74.7	21.1	72.6	–	81.9 at 5 years	60 months
Shi et al. [7]	188	52.7	85.1	78.2	29.9	73.4	75.9 at 5 years	–	40.5 months
Cui et al. [8]	25	52.3	88	64	–	80	–	–	36.5 months
Walsh & Bleiweiss [37]	80	58.8	90.6	70.3	59.1	72.3	–	–	–
Vingiani et al. [38]	49	52.7	87.8	69.4	18.4	69.4	–	89.8 at 6 years	6 years
Adrada et al. [39]	29	56^a^	82	61	43	62	–	–	–
Chen et al. [40]	100	50	46	27	–	84.8	63.3	59 at 5 years	60.1 months
De La Cruz et al. [41]	16	50.9	50	31.2	50	92.9	–	75	38 months
Luna-Moré et al. [42]	68	54.3	74.5	46.3	–	90.5	–	63	52.6 months
Middleton et al. [43]	14	50	25	12.5	–	–	70 at 5 years	–	57.6 months
Nassar et al. [44]	83	61	71	–	–	77	40	46	7 years
Paterakos et al. [45]	18	55	61.1	< 50	–	95.2	–	50 at 44 months	165.6 months
Pettinato et al. [46]	62	57	32	20	95	90	49	–	5.2 years
Yamaguchi et al. [47]	15	60.1	73	67	33.3	46.6	–	–	–
Zekioglu et al. [48]	53	52.5	68	61	–	68.8	–	72	56.5 months

*ER* estrogen receptor, *+* positivity, *PR* progesterone receptor, *HER2* human epidermal growth factor receptor 2, *RLN* regional lymph node metastasis, *DSS* disease-specific survival, *OS* overall survival, *NCDB* National Cancer Database, – not available or not reported

^a^Median or mean age

The IMPC patient characteristics in the present study differ in some ways from the IMPC patient characteristics reported in the literature. For example, the median age of presentation for IMPC patients, while similar to the SEER database analysis [3, 15], was older than the age at presentation reported in other IMPC patient series [2, 3, 5, 6]. In addition, we found higher rates of hormone receptor positivity than the rates in those series. As we know, ER positivity is associated with older age and longer OS of breast cancer patients as a whole [16, 17], which may explain the favorable survival outcomes for IMPC patients in the present study.

On multivariate analysis, ER positivity was associated with improved prognosis. This finding speaks to the growing focus in oncology on the molecular and biologic characteristics of disease rather than the clinical presentations and stage. The majority of patients in our analysis fall under the luminal A/B molecular subtypes (hormone receptor-positive, HER2-negative), which are associated with better outcomes than HER2-positive or triple-negative disease [18–20].

It is interesting to note that age < 50 years was associated with prolonged OS, as it has been previously observed that breast cancer patients who present at a younger age tend to have worse outcomes [21–23]. This finding may be unique to this particular subtype of breast cancer although several contributing and confounding factors may also be at play. In breast cancer as a whole, patients who present at a younger age are more likely to have more aggressive molecular subtypes, higher grade disease, and present at a more advanced stage than those at an older age [24–26]. As noted previously, the large majority of IMPC patients in our analysis had high rates of ER and PR positivity and therefore fall under the luminal A and B molecular subtypes, which may explain why the younger patients in our study did not have a worse prognosis. In addition, because the NCDB tracks only OS and not cause-specific or disease-specific survival, it is possible that patients older than 50 years had other comorbidities that affected the survival outcomes. Indeed, a Charlson–Deyo comorbidity score ≥ 1 was associated with short OS of IMPC patients on both univariate and multivariate analyses, which is consistent with the observations on breast cancer as a whole [27–29].

Another important and interesting finding of our analysis is that EBRT was associated with prolonged OS on univariate analysis. Importantly, the OS benefit was limited to patients receiving lumpectomy, and no OS benefit was observed among patients receiving mastectomy. Radiotherapy is well known to improve locoregional control and OS after breast-conserving surgery and mastectomy [30–33], but has not been studied specifically in IMPC. It is possible that, due to the high propensity of lymph node involvement in IMPC, EBRT may be important to provide good locoregional control and, subsequently, OS.

For breast cancer as a whole, the role of EBRT in nodal disease has evolved over time [28]. Given IMPC’s lymphotrophic nature, whether regional nodes should be included along with the standard whole-breast irradiation field is an important issue. Recent trials have highlighted prolonged disease-free survival with regional nodal irradiation (RNI) in patients with early-stage breast cancer [34, 35]. In addition, a meta-analysis conducted by the Early Breast Cancer Trialists’ Collaborative Group demonstrated that the survival benefit of postmastectomy radiotherapy with comprehensive lymph node coverage is not limited to patients with ≥ 4 positive lymph nodes, but also extended to patients with 1–3 positive lymph nodes [36]. Although EBRT likely plays an important part in the treatment of IMPC, the specific role of RNI in this subtype is still unclear.

Our study has several limitations due to its reliance on the NCDB. First, the retrospective nature of the study and all associated inherent biases must be acknowledged. The lack of central review of pathology specimens is another limitation; it is not clear what threshold level of micropapillary involvement was required for the samples to be flagged as IMPC in the database. However, previous studies have failed to find an association between the degree of micropapillary involvement and OS or lymph node involvement, suggesting that the presence of IMPC involvement (not the degree of involvement) is the most important factor in determining outcomes [2, 37]. The NCDB also does not include information of the receipt of targeted therapy. Finally, although the NCDB has information regarding the treatment delivered, it does not have information regarding the reasons for the delivery of each treatment. It is possible that patients who did not receive radiotherapy may have had a low Eastern Cooperative Oncology Group (ECOG) or Karnofsky Performance Status (KPS) score, and that the observed short OS in these patients was likely due to their underlying poor performance status and not the omission of radiotherapy.

## Conclusions

Although IMPC has a high propensity for lymph node metastasis, patients’ OS is comparable to the historical OS of IDC reported in literature. On univariate analysis, ≥ 4 positive lymph nodes, a Charlson–Deyo comorbidity score ≥ 1, and age > 65 years were associated with short OS. In contrast, receipt of EBRT and ER positivity were associated with prolonged OS. This study demonstrated a survival benefit of IMPC patients associated with EBRT, though this may be limited to patients receiving lumpectomy.

## Data Availability

The data that support the findings of this study are available from the NCDB but restrictions apply to the availability of these data, which were used under license for the current study, and so are not publicly available. Data are however available from the authors upon reasonable request and with permission of the NCDB.

## Abbreviations

IMPC: invasive micropapillary carcinoma
IDC: invasive ductal carcinoma
NCDB: National Cancer Database
OS: overall survival
ACoS: American College of Surgeons
ER: estrogen receptor
PR: progesterone receptor
HER2: human epidermal growth factor receptor 2
EBRT: external beam radiotherapy
HR: hazard ratio
SEER: Surveillance, Epidemiology, and End Results
RNI: regional nodal irradiation
ECOG: Eastern Cooperative Oncology Group
KPS: Karnofsky Performance Status
